# The Revolution in Migraine Genetics: From Aching Channels Disorders to a Next-Generation Medicine

**DOI:** 10.3389/fncel.2016.00156

**Published:** 2016-06-13

**Authors:** Simona Pellacani, Federico Sicca, Cherubino Di Lorenzo, Gaetano S. Grieco, Giulia Valvo, Cristina Cereda, Anna Rubegni, Filippo M. Santorelli

**Affiliations:** ^1^Clinical Neurophysiology Laboratory, IRCCS Stella Maris FoundationPisa, Italy; ^2^Molecular Medicine, IRCCS Stella Maris FoundationPisa, Italy; ^3^Don Carlo Gnocchi Onlus FoundationMilan, Italy; ^4^Genomic and Post-Genomic Center, C. Mondino National Institute of NeurologyPavia, Italy

**Keywords:** migraine, calcium channel, sodium channel, tripartite synapse, astrocyte, glutamate

## Abstract

Channelopathies are a heterogeneous group of neurological disorders resulting from dysfunction of ion channels located in cell membranes and organelles. The clinical scenario is broad and symptoms such as generalized epilepsy (with or without fever), migraine (with or without aura), episodic ataxia and periodic muscle paralysis are some of the best known consequences of gain- or loss-of-function mutations in ion channels. We review the main clinical effects of ion channel mutations associated with a significant impact on migraine headache. Given the increasing and evolving use of genetic analysis in migraine research—greater emphasis is now placed on genetic markers of dysfunctional biological systems—we also show how novel information in rare monogenic forms of migraine might help to clarify the disease mechanisms in the general population of migraineurs. Next-generation sequencing (NGS) and more accurate and precise phenotyping strategies are expected to further increase understanding of migraine pathophysiology and genetics.

## Introduction

The channelopathies are a heterogeneous group of neurological disorders that result from genetic dysfunction of ion channels located in cell membranes and organelles. Similarly to ion pumps and transporters, ion channels are highly selective and coordinate ion fluxes during the generation of action potentials, or following neurotransmitter release, in the nervous system and muscles (Spillane et al., 2016). Their dysfunction may therefore impair neuronal excitability and synaptic transmission, thus constituting a key pathophysiological element of a wide range of disorders.

Generally, the symptoms of channelopathies appear early in life and are typically paroxysmal or episodic. Defects in a single channel may lead to different neurological manifestations, e.g., seizures, paroxysmal movement disorders/periodic paralyzes, and migraine. However, despite the variable presentations, certain trigger factors (i.e., sleep, stress, hormonal fluctuations), patterns of age dependence of manifestations, and treatment modalities may overlap, suggesting the existence of common pathogenic substrates. Conversely, defects in different ion channels, or transporters, can often underpin the single neurological picture. It is therefore difficult to predict the clinical consequences of ion channel dysfunctions, and to establish clear pathophysiological explanations in episodic neurological channelopathies. The reason why defects in single channels can lead to seizures, episodic ataxia, movement disorders or migraine, or to a combination of these, is only partially understood and possibly dependent on diverse molecular mechanisms that affect channel function (i.e., gain- or loss-of-function effects of mutations), and on the specific neuronal circuitry involved.

In this mini review, we focus on the complex pathophysiological relationship underlying migraine disorders, in which an array of genetic and environmental components strongly contributes to variable individual susceptibility and clinical manifestations (i.e., migraine with aura [MA] or without aura [MO]). Indeed, largely because of the phenotypic heterogeneity and genetic pleiotropy and variability of migraine disorders (de Vries et al., 2009), investigation of the common forms of migraine has, to date, provided only limited insight into the underlying genetics and pathophysiology. Studies on rarer monogenic forms of migraine (termed familial hemiplegic migraine [FHM] syndromes), on the other hand, have identified new genes pinpointing fundamental disease mechanisms that possibly also contribute to the common forms of migraine in the general population.

## Migraine: Clinical and Pathophysiological Aspects

Migraine affects about 15% of the general population, and women three times more often than men (Launer et al., 1999; Jensen and Stovner, 2008). It is characterized by episodic and disabling attacks of headache, often accompanied by nausea, vomiting, photophobia and/or phonophobia, which may, or may not, be preceded by an aura. Aura is a transient neurological symptom, lasting 5–60 min, that usually includes visual, sensory and/or aphasic features. Even though MA and MO are considered distinct disorders, increasing evidence suggests that the two conditions are, in fact, variable clinical expressions of substantially the same genetic disease. Indeed, the fact that the prevailing migraine form in a single patient might vary over time suggests that the main pathophysiological pathways are essentially the same in MO and MA, and that external modulating factors might favor the switching on/off of one of the two migraine types (Ferrari et al., 2015).

Auras are likely caused by the phenomenon of cortical spreading depression (CSD), namely a wave of neuronal and glial depolarization that starts in visual cortical areas and moves slowly (2–6 mm/min) over the cortex (Lauritzen, 1994). CSD is thought to be the consequence of noxious stimuli that alter the neuronal environment, leading to glutamate-induced toxicity (Kramer et al., 2016). Glutamate activates cation currents, particularly through the N-methyl-D-aspartate receptors, leading to near breakdown of neuronal transmembrane ion gradients (Ca^2+^, Na^+^, Cl^-^, and K^+^). This loss of potential, which is normally reinstated by Na^+^/K^+^ pumps, is not recovered immediately, resulting in long-lasting inhibition of spontaneous and evoked neuronal activity (Dreier and Reiffurth, 2015). Although the mechanism of CSD has been extensively investigated in animal models (Charles and Baca, 2013), experimental evidence in humans is still scarce. In MA, functional magnetic resonance imaging findings have revealed a local increase in blood oxygen level-dependent signals, which were found to spread through the visual cortex at a rate similar to what is seen in experimentally induced CSD in animals (Hadjikhani et al., 2001). Magnetoencephalography studies have also shown that large cortical areas are activated in spontaneous and visually induced migraine auras, producing a spreading depression-like neuroelectric event that may be likened to CSD (Bowyer et al., 2001).

The pain in migraine headache results from activation of the trigeminovascular system (Noseda and Burstein, 2013). Indeed, signals from activated nociceptors located on large cranial vessels and the dura mater are transmitted to the trigeminal bipolar neurons, and further relayed, through extensive connections with brainstem regions (i.e., the periaqueductal gray and locus coeruleus), to thalamic and cortical areas, ultimately producing the sensation of pain (Ferrari et al., 2015).

Multiple evidences suggest that CSD might not only cause migraine auras, but also, by itself, trigger the mechanisms underlying the headache and associated symptoms. These mechanisms consist mainly of the release, by neurons, glia and vascular cells, of pro-inflammatory peptides, such as substance *P* and calcitonin gene-related peptide, but also adenosine triphosphate (ATP), glutamate and potassium, and the resulting local increase in neuroactive inflammatory mediators and sensitization of pain-relevant brainstem regions (Zhang et al., 2007; Levy, 2012). The opening of neuronal Panx1 channels in response to the CSD stimulus also helps to trigger an inflammatory cascade by releasing HMGB1 proteins, which activate neighboring astrocytes leading to sustained release of inflammatory mediators (Karatas et al., 2013). Although definitive proof is lacking, drugs preventing CSD may be effective in treating migraine attacks (Costa et al., 2013). Pain is only the tip of iceberg of a complex chronic disease in which several molecular mechanisms lead to increased susceptibility to CSD (Antal et al., 2008) and the release of soluble mediators, and thus to long lasting neuronal sensitization, amplified nociceptive signaling by trigeminal sensory neurons, and stable neuroinflammatory tissue responses (Franceschini et al., 2013). Indeed, clinical and neurophysiological studies have confirmed that individuals suffering from migraine display chronic hypersensitivity to sensory stimuli or abnormal processing of sensory information (Aurora et al., 2007; Vecchia and Pietrobon, 2012), which may be reflected in more frequent premonitory symptoms (e.g., speech/reading difficulties, sensory hypersensitivity) preceding the attacks (Pietrobon and Moskowitz, 2013).

## Familial Hemiplegic Migraine

Molecular insights into the rare monogenic FHM syndromes have highlighted the central role of calcium (Ca^2+^) and sodium (Na^+^) channels, and of sodium-potassium (Na^+^/K^+^) ATPase, in the etiology and pathophysiology of migraine. In FHM, migraine attacks are associated with transient hemiparesis, lasting minutes to hours or days, or alternatively may present as episodes of “regular” MO or MA without major motor weakness. Patients may also suffer from a variety of symptoms that include cerebellar ataxia, seizures and even mild head trauma-induced brain edema that can be fatal (Kaja et al., 2010). Three FHM genes have been identified: *CACNA1A* (FHM1; Ophoff et al., 1996), *ATP1A2* (FHM2; De Fusco et al., 2003), and *SCN1A* (FHM3; Dichgans et al., 2005).

*CACNA1A* codes for the alpha subunit of the neuronal voltage-gated Ca^2+^ channel Ca_v_2.1 (Diriong et al., 1995). Ca_v_2.1 channels are predominantly expressed at the presynaptic terminals of glutamatergic and GABAergic neurons in the cerebral cortex, trigeminal ganglia, brainstem nuclei and cerebellum (Catterall, 1998), where they play a crucial role in neurotransmitter release. The clinical features of Ca_v_2.1 channelopathies range from pure FHM1 to forms that include episodic or progressive ataxia (Jouvenceau et al., 2001; Imbrici et al., 2004) and seizures. In FHM1, *CACNA1A* mutations typically lead to a gain of Ca_v_2.1 channel function, although in model organisms this seems have the effect of enhancing only glutamatergic neurotransmission, whereas inhibitory synapses remain unaffected (Tottene et al., 2009). This differential effect at excitatory and inhibitory synapses suggests that altered regulation of cortical excitatory-inhibitory balance may be a likely pathomechanism in FHM1. The gain of Ca_v_2.1 channel function may indeed favor glutamate release, and consequently the induction and propagation of CSD (Vecchia and Pietrobon, 2012; Pietrobon and Moskowitz, 2013).

The second FHM gene (FHM2), *ATP1A2*, encodes the alpha-2 subunit of a Na^+^/K^+^ pump (De Fusco et al., 2003). This catalytic subunit utilizes ATP hydrolysis to exchange Na^+^ ions (leaving the cell) for K^+^ ions (entering the cell) and is present in the membrane of astrocytes at tripartite synapses, where it contributes to K^+^ and glutamate re-uptake. More than 30 FHM2 mutations have been identified (de Vries et al., 2009) and associated with pure disease (De Fusco et al., 2003; Riant et al., 2005; Vanmolkot et al., 2006), or with a combination of FHM and cerebellar ataxia (Spadaro et al., 2004), recurrent encephalopathy (Ducros et al., 2001; Spacey et al., 2005), impaired hearing and vertigo (Jurkat-Rott et al., 2004), or epilepsy (Roth et al., 2014). Some *ATP1A2* mutations have also been associated with non-FHM phenotypes, such as basilar migraine (Ambrosini et al., 2005) or common migraine (de Vries et al., 2009). Defective function of glial Na^+^/K^+^-ATPase at tripartite synapses may interfere with glutamate clearance by astrocytes, leading to increased cortical excitatory neurotransmission which facilitates CSD.

The third FHM gene (FHM3), *SCN1A*, encodes the alpha-1 pore-forming subunit of the neuronal voltage-gated Na^+^ channel Na_v_1.1 (Dichgans et al., 2005). Voltage-gated sodium channels have a crucial role in cellular excitability and are essential for the initiation of action potentials in the brain. Mutations in *SCN1A* are associated with a wide spectrum of epilepsy phenotypes (e.g., severe myoclonic epilepsy; Marini et al., 2007; Dravet and Oguni, 2013). More rarely, *SCN1A* mutations lead to pure FHM (Dichgans et al., 2005; Vanmolkot et al., 2007), or to FHM associated either with generalized seizures (Castro et al., 2009), or with a stereotyped phenotype (elicited repetitive transient daily blindness) that suggests a retinal form of spreading depression (Vahedi et al., 2009; Fan et al., 2016). Epileptogenic Na_v_1.1 mutations cause loss of channel function of variable degrees, leading to reduced Na^+^ currents in GABAergic inhibitory interneurons (Yu et al., 2006), thus defining an interneuron-specific generalized defect in action potential initiation which results in multisystem disinhibition and network hyperexcitability (Hedrich et al., 2014). Mutated Na_v_1.1 channels in FHM3 instead exhibit a broad range of abnormalities, including gain of function and partial or complete loss of function, confirming the complex relationship between clinical and biophysical phenotypes in *SCN1A*-related pathology (Kahlig et al., 2008). Regardless of the molecular mechanism, however, the high-frequency firing of mutant Na_v_1.1 channels, by producing a rise in extracellular K^+^ concentration and consequent further depolarization, may enhance the release of glutamate and sustain CSD mechanisms.

Taken together, the three different forms of FHM indicate the existence of a main pathophysiological pathway that, starting from excessive neuronal release of glutamate (*CACNA1A*), impaired glutamate reuptake by glial cells (*ATP1A2*), or enhanced glutamatergic activity due to impaired GABAergic inhibition (*SCN1A*), ultimately leads to altered glutamatergic neurotransmission, with consequent neuronal hyperexcitability and increased susceptibility to CSD (Ferrari et al., 2015). The three major genes, however, do not account for all affected cases, and at least three additional genes (*SLC1A3*, *PRRT2* and *SLC4A4*) have been suggested, albeit on the basis of limited evidence, to be associated with FHM in a minority of cases. Notably, defects in all of these genes lead to enhanced excitatory neurotransmission and cortical excitability. Mutations in *SLC1A3*, encoding the excitatory amino acid transporter 1 (EAAT1; Jen et al., 2005), cause decreased glutamate reuptake, whereas *PRRT2*, through defective interaction with SNAP25 and GRIA1 proteins (Li et al., 2015), affects the glutamate signaling pathway and results in increased glutamate release. Finally, the sodium bicarbonate cotransporter NBCe1 (*SLC4A4*) may derange synaptic pH regulation in astrocytes, leading to neuronal hyperexcitability predisposing to migraine (Suzuki et al., 2010).

## Main Genetic Issues in the Study of Migraine

Migraine is a multifactorial disorder resulting from complex interactions between multiple predisposing genes and environmental factors (Russell et al., 1995; Mulder et al., 2003). The latter include hormone fluctuations, and this may explain the increased prevalence of migraine in females, and its variability across the individual life span (MacGregor, 2004). The clinical presentation is also variable, making the pathogenesis of migraine particularly difficult to unravel. Furthermore, it is not clear whether, from the perspective of genetic study designs, the two forms (MO and MA) should be considered the same disease, given that findings from epidemiological and clinical studies are still conflicting (Russell et al., 2002; Ligthart et al., 2006). Many clinical aspects contribute to the extreme variability of migraine phenotypes: for example, the severity and frequency of the attacks, the attack triggers, and the neuropsychiatric comorbidities possibly involved (depression, epilepsy, etc.) are all highly variable. This strong heterogeneity, together with the lack of any genetic biomarker, makes it difficult to stratify patients for genetic studies and consequently to identify strong genotype-phenotype correlations. Previous linkage analyses on large pedigrees, and screening of candidate genes, including more than 150 ion transporter genes (Nyholt et al., 2008), in several thousand migraineurs were largely unsuccessful (de Vries et al., 2009). Similarly, testing of the three major FHM genes in patients with common migraine has shown no evidence to support their involvement in the disorder. It is indeed possible that disease risk in common migraine may be conferred by multiple genes and their variants (each with a small effect size) that control neurotransmitter and ion pathways through complex interactions and regulatory mechanisms (Eising et al., 2013b). Moreover, none of the three major FHM genes has been identified in unbiased genome-wide association (GWA) studies, which until now have constituted the most robust approach for identifying genetic factors underlying complex disorders (Spain and Barrett, 2015). Overall, GWA studies have uncovered 13 susceptibility loci that involve a set of genes clustering into clear pathways likely related to migraine (Freilinger et al., 2012). Notably, several of these genes (i.e., *MTDH*, *LRP1*, *PRDM16*, *MEF2D*, *ASTN2*, *PHACTR1*, *FHL5*, *MMP16*) are involved in glutamatergic neurotransmission and synaptic function/development, whose impairment may therefore be considered a main dysfunctional mechanism underlying susceptibility to common forms of migraine. Pain-sensing mechanisms, metalloproteinases and vessel metabolism seem likely to be additional migraine-related pathways (Tolner et al., 2015). The strong association between candidate genes emerging from GWA studies and glutamate metabolism is in line with evidence from FHM, which suggests that impaired glutamatergic neurotransmission is a key disease mechanism underlying the abnormal cortical excitability that favors the initiation and propagation of CSD and the recurrence of migraine attacks. However, none of these candidate genes can conclusively be regarded as a genetic biomarker of the disease; each has limited predictive value given their small effect size (Di Lorenzo et al., 2015). It is possible that multiple gene variants affecting protein-to-protein interactions play an important role in disease mechanisms in specific clinical conditions. For example, analysis of GWA study data, looking for specific disease-relevant functional gene sets, i.e., lists of genes related to glial metabolism or synaptic function (Eising et al., 2015), has disclosed a role for astrocyte- and oligodendrocyte-related genes in MO and MA.

**Figure 1:**
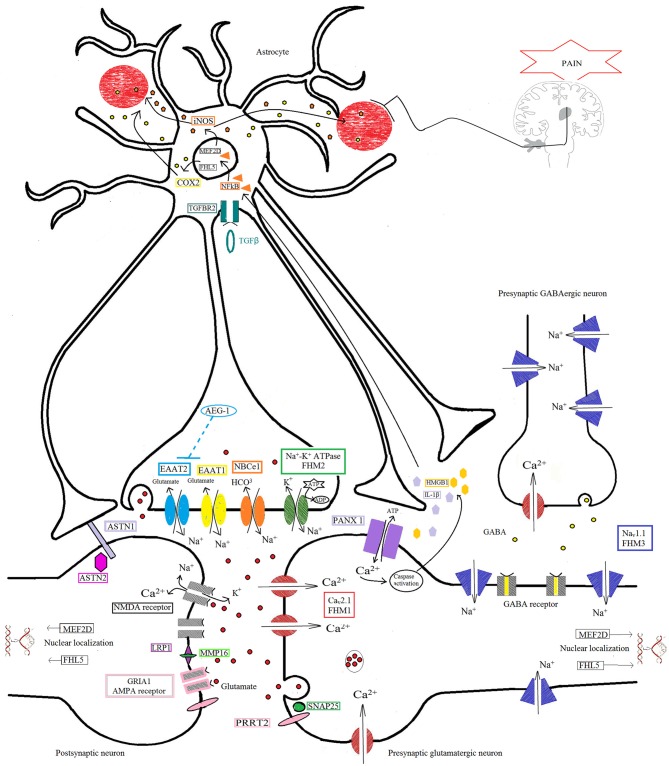
**Protein pathway driving the migraine process at the tripartite synapse.** The illustration depicts different proteins at the tripartite synapse possibly involved in glutamatergic dysfunction in migraine (see text for details). Ca_v_2.1 (*CACNA1A*; red) dysfunction at presynaptic terminals of glutamatergic neurons leads to altered Ca^2+^ influx and enhanced glutamate release by vesicles into the synaptic cleft, favoring the activation and propagation of cortical spreading depression (CSD) in familial hemiplegic migraine 1 (FHM1). Na^+^/K^+^-ATPase (*ATP1A2*; green) at the astrocyte plasma membrane utilizes ATP hydrolysis to exchange Na^+^ for K^+^ ions, generating a Na^+^ gradient that helps to modulate the glutamate re-uptake by glial excitatory amino acid transporter 1 (EAAT1; *SLC1A3*; yellow) and EAAT2 (*SLC1A2*; cyan). Loss-of-function of Na^+^/K^+^-ATPase (FHM2), as well as of EAAT1, slows the clearance of glutamate leading to increased cortical excitability that favors the initiation and propagation of CSD. The activity of EAAT2 also contributes to glutamate clearance, and is downregulated by mutations in astrocyte elevated gene-1 (AEG-1) (*MTDH*; dashed line), one of the candidate genes emerging from genome-wide association (GWA) studies. Na_V_1.1 channels (*SCN1A*, FHM3; purple) are essential for the generation and propagation of action potentials. FHM3-associated mutations can reduce firing of inhibitory interneurons, or accelerate the recovery of the channel after fast inactivation, causing high-frequency firing of presynaptic glutamatergic neurons. PRRT2 (pink) also affects the glutamate signaling pathway, through defective interaction with SNAP25 (forest green) and the ionotropic glutamate receptor AMPA1 (termed GRIA1; gray with pale pink border), resulting in increased glutamate release. Defective membrane expression of the Na(^+^)-HCO(3)(-) cotransporter NBCe1 (*SLC4A4*; orange) may affect the uptake of HCO3- into astrocytes leading to altered activity of pH-sensitive NMDA receptors (gray). Both AMPA and NMDA receptors are also directly modulated by LRP1 (dark pink), which is cleaved by a metalloproteinase that is encoded by another migraine-susceptibility gene, MMP16 (fluorescent green). Synaptic activity is also influenced by other proteins thought to contribute to migraine pathophysiology, such as the nuclear transcription factors MEF2D and FHL5, the serine-threonine kinase TGFBR2 (aquamarine), and ASTN2 (fuchsia), a protein related to ASTN1 (pale lilac) and thought to influence neuronal migration. All these mechanisms, when defective, may affect the glutamate signaling pathway, possibly leading to neuronal hyperexcitability predisposing to migraine. The illustration also shows the pathway that starts from the CSD-driven opening of PANX1 channels (lilac) and triggers the inflammatory cascade and subsequent trigeminovascular sensitization. Signaling to PANX1 leads to caspase 1 activation that, in turn, stimulates the release of high-mobility group box 1 (HMGB1) proteins and the activation of the transcription factor nuclear factor κB (NF-κB) in astrocytes. This may lead to local increase in vasoactive inflammatory mediators and sensitization of pain-relevant brainstem regions.

It is to be hoped that thanks to the advent of novel and cost-effective genetic technologies, known collectively as next-generation sequencing (NGS), it will be possible to overcome some limitations of the more traditional approaches used to investigate the genetic basis of migraine. NGS will serve not only as a tool for identifying new genes responsible for monogenic forms of the disorder (i.e., FHM), but also, quite probably, for identifying low-frequency variants that have moderate effects in more common forms of migraine. The combination of data from monogenic migraine and GWA studies, will make it possible to pinpoint the biological systems crucially involved (e.g., glutamatergic neurotransmission and metabolism at the tripartite synapse, Figure 1), and to use this information to design specific customized gene panels allowing thorough investigation of the contribution made by each single molecular player to the dysfunctional protein networks underlying this complex polygenic disease.

## Conclusions and Future Directions

**Figure 2:**
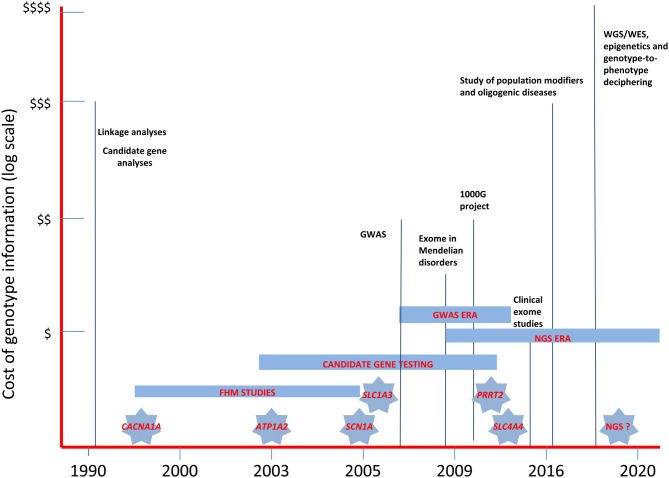
**The landscape of past and near future genetic research in migraine headache disorders.** The diagram illustrates the relative costs of the molecular methodologies employed in the study of migraine genetics and the timing of the discovery of the major genes involved in FHM and in related disorders. WES, whole-exome sequencing; WGS, whole-genome sequencing; GWAS, genome-wide association studies; NGS, next-generation sequencing.

The emerging NGS techniques are seen as the most promising resource for overcoming gene-finding problems in future migraine genetic research. The various approaches tried to date, using linkage, candidate gene and GWA studies (Figure 2), have not been sufficient to unravel the complex genetic background of MA and MO. In addition, other mechanisms, such as gene-gene or gene-environment interactions and epigenetics, further complicate the already complex picture of the heritability of migraine syndromes (Rudkjobing et al., 2012), suggesting that genotyping data need to be integrated with the results of deep clinical stratification, gene expression data, and proteomics/metabolomics studies in order to fully understand the effects of genetic variability (Bras et al., 2012). The process of deep phenotyping is expected to be a crucial tool for future research in migraine genetics. Focusing genetic analyses on groups with more homogeneous presentations will help in investigating the function and pathogenic relevance of gene variants emerging from NGS studies, strongly increasing the power of genetic information and the strength of genotype-phenotype correlations (Hennekam and Biesecker, 2012) and paving the way for more personalized/“precision” medicine (Zhang et al., 2016). Evaluation of migraine comorbidities, in particular, should be regarded as a pivotal part of the stratification process. It is well established, for example, that adults and children with migraine may have increased susceptibility to seizures (Rajapakse and Buchhalter, 2016). Identifying this potential comorbidity may allow efforts to dissect the genetic basis of the condition to be targeted toward specific sets of genes that may have a role in both migraine and epilepsy pathophysiology, such as the ion channels enhancing excitatory neurotransmitter release (i.e., Ca_v_2.1) or dendrite neuronal excitability and firing (Na_v_1.1), and their molecular interactors. The use of metabolic parameters will also help the stratification process, possibly favoring the discovery of new genetic biomarkers based on NGS analyses. Gene expression alterations could be used as markers of epigenetic mechanisms (DNA methylation, histone tail modifications, noncoding RNA metabolism) thought to play a role in the development of migraine (Eising et al., 2013a). Finally, data from studies of proteomics and metabolomics might make it possible to define the full metabolic profile of individuals suffering from migraine, fostering efforts to arrive at a phenotypic dissection at molecular level. Although we still do not know whether metabolic changes can be detected in peripheral fluids of migraine patients, data on animal models are promising. Indeed, Ca_v_2.1 transgenic mice have shown measurable metabolic changes in plasma after experimentally induced CSD (Shyti et al., 2015). Defining clinical phenotypes and detectable biomarkers in humans might enable a better understanding of the molecular pathways involved in migraine, and thus allow more accurate understanding of the bulk of data emerging from NGS.

To date, very little research using NGS methods in migraine has been published, and that which can be found is limited to the sequencing of very few familial cases (Nagata et al., 2014; Jiang et al., 2015). There are, indeed, some technical problems that limit the use of NGS in complex polygenic disorders (Topper et al., 2011), and translation of data into diagnostic information often requires further validation through functional assays, even using unanticipated new tools (Doğanli et al., 2013). As already established in relation to other neurodevelopmental disorders (e.g., autism, intellectual disability, etc.; Hoischen et al., 2014), targeted resequencing approaches may be a valid strategy for reducing the costs and improving the specificity of analyses. The application of pathway-focused large gene panels or biomarker-driven genomic investigations, combined with a stringent endophenotype-oriented approach, may allow a deeper assessment of the role of specific proteins presumably involved in migraine pathomechanisms (for example, those belonging to the dysfunctional pathway at the astrocyte-neuron synaptic cleft, Figure 1). This could also lead to the discovery of new biologically relevant checkpoints in the pathways crucially involved in aura and pain mechanisms underlying migraine disorders. As the costs associated with genome-scale sequencing progressively fall, and new tools for high-throughput functional assays are developed, NGS techniques will gradually become a more feasible clinical option for the decoding of complex polygenic conditions such as migraine, revealing previously unexpected opportunities for personalized medicine.

## Author Contributions

All the authors have contributed substantially to the writing and revising of the manuscript. SP, FS, and FMS participated in the conception and design of the work, collected the literature, prepared the figures and wrote the manuscript. CDL, CC, GSG, GV, and AR reviewed and edited the manuscript, and approved the final version.

## Funding

Research in our laboratories is partially supported by grants from the Italian Ministry of Health (Ricerca Corrente to FMS) and Telethon-Italy (http://www.telethon.it/en; Grant no. GGP11188 to FS).

## Conflict of Interest Statement

The authors declare that the research was conducted in the absence of any commercial or financial relationships that could be construed as a potential conflict of interest.

## Abbreviations

MA: migraine with aura
MO: migraine without aura.
